# Government (Teaching) and Municipal (Non-Teaching) German Hospitals


**Published:** 1912-12-07

**Authors:** Henry Burdett


### GOVERNMENT (Teaching) and MUNICIPAL (Non-Teaching) GERMAN HOSPITALS.*

By SIR HENRY BURDETT, K.C.B.

### VIII. The Classification of In-patients and Conditions of Admission.

Great interest has centred round the admission and treatment of in-patients in the municipal hospitals in Germany. The regulations in the larger cities, though they may differ in some details, for practical purposes follow mainly the same lines. This becomes apparent when we examine the regulations for municipal hospitals in Berlin, Hamburg, Dresden and Munich, to name only four great cities as fairly representative of the system pursued. Every patient seeking admission is relegated at once to his class. It is essential to keep clearly in mind that the whole plan of admission to municipal hospitals is based upon the cardinal principle that every single patient, quite irrespective of his social position, shall pay or be paid for during the whole time of residence in the hospital. Thus the "Weary Willies" and all members of the casual population without means are paid for at a fixed rate from some source. The term "sick poor" in the German meaning relates to all such persons, and to all who are not members of a Kasse or who have not the means to defray the cost of their treatment in a hospital. People who are found helpless or are brought to the hospitals by the police, subject to exclusion as explained later, are admitted to the cheapest wards. Admission is governed by the physical needs of the patient, and is at the discretion of the receiving practitioner in charge of the admission department, subject only to any regulation to the contrary contained in the laws of the magistrates or Hospital Commission.

In cases of emergency poor-law practitioners are authorised to send patients direct to the hospitals, and admission is immediate if the urgency of the case is expressly certified in the practitioner's order, which has to be stamped and signed by the poor-law medical officer. All patients retained in municipal shelters, homes, chronic hospitals, etc., are admitted to the municipal hospital on the order of the manager of any such establishment. In all other cases an admission order signed by the poor-law medical officer has to be stamped and signed also by the President of the Poor-Law Commission or the manager. The officials and employees of the municipality, though they may reside outside the city boundaries, poor-law pupils of the municipality, and persons whose maintenance is required by law to be at the cost of an authority within the municipal area, are admissible without question. The same rule applies to persons who have been sent by a municipal authority for hospital treatment from outside the municipal area.

Kasse patients are admitted under contracts made between the hospital authorities and the managers of each Kasse. These agreements usually allow a reduction, on the tariff rates, of from 16 to 20 per cent. in such cases. The Kasse patients may, if they choose, pay an additional sum to entitle them to be treated in a higher class than that to which Kasse patients as a rule are relegated. Paying patients apply for admission on a doctor's certificate stating the nature of the case and its urgency, but the date of their admission is governed by the pressure upon the beds. The pay-patient may have to wait for a bed in a pay-ward, whereas the patients seeking admission to the third, or lowest, class of ward are usually taken in without delay. All patients except paying patients receive body linen (with the exception of headgear) and the necessary hospital dress from the hospital stores. All the washing is done in the hospital laundry for this class of patient. Paying patients are allowed to wear and bring their own clothing, providing they conform to the regulations of the hospital and the orders of the doctor. They must provide a plentiful supply of body linen, and in case they suffer from a contagious disease they are not allowed to have their washing done outside the hospital, nor are they allowed to provide their own beds and bed linen without special permission.

It may be well to mention here that the scale of payments, which varies from two classes, as at Dresden—(1) paying patients and (2) all other patients—to four classes in the Hamburg hospitals —*i.e.*, (1), (2), and (3) paying patients and (4) ordinary patients and children under ten—provides that residents and ratepayers of the city are received at a lower rate than non-residents. Thus at Hamburg the payments for paying patients vary from 4 marks per day to 12 marks per day for residents in Hamburg to from 6 marks to 15 marks per day if non-residents. In the same way the payments for the fourth class of patients are, if residents, 2.50 marks per day for adults and 1.50 marks per day for children under ten, and for non-residents 3.50 marks per day for adults and 2.25 marks for children under ten.

### Patients Excluded from Admission.

The regulations contain provisions excluding from admission certain persons. These include persons who are held to be ineligible on account of their state of health, as prescribed by the Imperial and Prussian laws, and the regulations for the administration of such laws; persons under sentence; mental patients, with an exception in Berlin in favour of patients forwarded by the city mental hospitals under existing regulations; violent patients; persons who at the date of their application are non-residents of Berlin, unless sent in through the poor law. Thus in the case of prisoners, mental patients, and non-residents admission is not to be refused when it might cause serious and immediate danger to the

* Previous articles of this series appeared in our issues of Oct. 12, 19, 26, Nov. 2, 9, 23, and 30.

life and health of the patients. When a person is brought in dead the rule is that the body should immediately be returned to those who brought it, or, should this not be possible, it is to be passed on to the mortuary.

Patients Cannot Select a Particular Hospital.

The rules for sending patients to the Berlin hospitals lay down pretty clearly the particular hospitals to which residents in certain districts are to apply. The admission of patients to each of the municipal hospitals is provided for by special rules for the sending of patients to the Berlin hospitals and sanatoria. The only exception to these rules is when the assigned hospital is full, or when the refusal to admit would cause danger to the life or health of the patient. Whenever a hospital refuses a patient admission on any ground, if he is suffering from an infectious disease, the hospital must arrange for the transfer of such patient by ambulance to a hospital where there is room. In other cases the refusing hospital must render to the patient every assistance with a view to secure his immediate admission elsewhere. We can well understand from our visits to the various hospitals that this rule may be very unpopular with the more intelligent residents in a city like Berlin, for instance, where the difference in the quality and the character of the hospital accommodation provided in certain municipal hospitals, compared with others, varies materially. An attempt, we believe, is made to meet such objections by anticipation through classification—that is, one hospital or more is set aside for septic and chronic cases, and the remaining hospitals, which are generally open to residents in Berlin, are maintained as far as possible at a uniform standard of efficiency.

Payments in Advance.

The whole hospital system in Germany is strict business, a fact which gives it a great advantage, and has tended so far to secure uniform efficiency. Money makes the mare to go, and all municipal hospitals in a great city should, under proper management, have equal financial strength. It is not surprising, therefore, to find the rules in several cities provide that municipal hospital patients shall make a deposit of at least one month's maintenance charges in advance. Thus, if a patient admitted to a pay ward or his friends fail to pay each week the maintenance charges fixed in the tariff, when the arrears of such payments relate to the whole of the current month and requests for payment remain fruitless, the patient is relegated to the pauper class and charged for on a pauper basis. The same rule governs exceptional cases where patients may have been admitted without advance payments, or where a Kasse fails to accept the liability for the cost of maintenance of one of its members. Another noteworthy rule is that non-residents in Berlin, who are admitted to a municipal hospital in that city, are charged the price demanded in their own municipality for outsiders, in so far as it is higher than the tariff at the Berlin municipal hospital. No advance payments are received from patients forwarded by an authorised institution—*i.e.*, a municipal authority, the Sickness Kassen and its unions, insurance societies, the Contributory Society of Berlin Servants, or in the case of patients admitted by order of the police as infectious cases. Even in the case of admission by order of authorised institutions the order must contain acceptance of full liability for the cost of maintenance without any qualification whatever.

Some municipal hospitals admit free patients, the cost of whose maintenance is defrayed out of endowments under their control. Thus in Dresden the municipality has a Town Hospital Fund for the benefit of the inhabitants of that city, out of which the cost of maintaining a number of free or partly free beds for needy persons, not yet under the care of the poor law, is defrayed by the administrators of the hospital. Another rule is that patients suffering from diseases caused by their own fault are not eligible for admission to free beds.

Formalities, Extras, and Inclusive Terms.

It is usual for an applicant for admission to produce a statement of personal and family circumstances with his address; written evidence that the applicant is known at the police station of the district, for purposes of identification, wherever possible; a written medical certificate of the nature of the patient's malady; and in the case of servants a written identification from the family in whose service they are, with proof of membership of the servants' insurance society. When a patient wishes for private and better furnished rooms—first class—he should inquire at the hospital if such a room is available before setting out. The hospital authorities do not guarantee that first-class patients will find a room immediately at their disposal. The tariff rates paid by patients of the lower class—*i.e.*, in the general wards—include food, living, nursing, medical treatment, medicines, and ordinary requirements. In the first class, medicines, fees for physical and medical baths and ordinary requirements are charged as extras; in both classes, spectacles, trusses, Röntgen treatment as per tariff, special medicines, consultations, reports and examinations, and ambulance expenses are charged as extras. No medicines may be brought in from outside. In the first class the fees for operations, for treatment by specialists, for special service and special food, as agreed or settled by the tariff, have as a rule to be deposited in advance. A reduction of 50 per cent. on the scale fees is sometimes allowed for second-class patients whose incomes do not exceed 3,400 marks, and a similar allowance may be made to residents in a city when suffering from notifiable contagious disease. All such requests have, however, to be made before admission to the administration or the authorities in writing on printed forms provided for the purpose. The administration declines all responsibility for articles of jewellery, money, and valuables, unless they are left in charge of the cashier to be placed in the hospital safes. The hospitals as a rule refuse to give information by telephone concerning patients; if a patient becomes seriously worse information is at once given to the relatives or friends without communication from them. Only grown-up relatives or friends are as a rule admitted as visitors; in the case of patients in the second class visitors are restricted to Sundays and Wednesdays from 2 to 4 p.m., but the friends of first-class patients may visit them daily in the morning and in the afternoon, unless the authorities or the doctors in attendance decide otherwise. When patients in the first class make unreasonable demands upon the arrangements or the attendance or both, an extra charge is added to the tariff rates in proportion to the trouble thus caused. A patient in the first class has a separate room, may have a special nurse, and may invite a friend to stay with him and share his room. In the second class of paying patients the patient must share his room with another patient to be selected by the doctor. At Hamburg, in the third class—that is, the lowest rate of paying patients as opposed to ordinary patients—there are five beds in each room. In Hamburg and other cities patients are never fetched by the hospital, and a patient or his relatives must engage an ambulance carriage where necessary.

The inclusive terms are as a rule (subject to reduction in certain cases already intimated for the poorer class of Kasse patients) 3 marks a day if resident in the town, and 3.50 marks to non-residents. In the lower class of paying patients the rates range from 4 to 7 marks per diem for residents and from 6 to 10 marks for non-residents. In the first class or highest grade of pay-patients the scale for maintenance may be taken as 12 marks per day for residents and 15 marks per day for non-residents.

### Security for Payments.

When it was determined to admit a higher class of paying patients into separate pavilions the management proceeded with caution, and only provided a limited number of beds and restricted accommodation for the higher-class patients. If we may judge by the comparative unattractiveness of the earlier pay pavilions, and the relative comfort and attraction of the later ones, in German hospitals, we must conclude that the policy of making provision for all classes of patients, and especially for the higher classes, at first was regarded probably as an experiment. Be this as it may, the provision for the higher class of paying patients has fulfilled a felt want and proved successful and popular. It is indeed an object lesson for us in Great Britain, where we stand at the edge of a new departure in hospital administration, which will, we hope, result in all hospitals of importance having special pavilions and accommodation for at least two classes of pay-patients above those treated in what have heretofore been free wards. These classes should be (1) where patients will pay 12s. a day and upwards, and (2) where patients will pay 6s. to 8s. a day.

The policy of the German hospitals to prevent all loss by non-payment of dues from patients makes the rules provide security for the maintenance expenses in all cases. In some cases the security is stated to be a deposit of 100 or 50 marks, according to the class of patient; in others the security is the equivalent of a sum equal to one month's or thirty days' hospital maintenance, according to the tariff charged for patients in each class. When the sum due each week by such patients is not paid, providing the deposit is exhausted, the patient may be continued on a pauper basis, as already explained above. In Berlin the municipality refuses all responsibility for the safety of valuables, but, when these are placed in the care of the hospital, the municipality pays itself, as occasion may arise, for the care and maintenance expenses incurred, out of moneys handed to it for safe keeping by the patient. When a savings-bank or other book relating to securities is given up for examination or other purpose, the municipality regards this act as authority to collect the withdrawal and pay the expenses incurred by the patient at the hospital out of the proceeds, in so far as an advance payment has not been made or is insufficient. Any articles of the patient which may be pledged may be disposed of subsequently at the discretion of the municipality either privately or by auction. In such cases a notification to the patient debtor is not necessary. These regulations throw much light on the position of the patient admitted to the lower-grade wards, at any rate in German hospitals. We say "lower-grade" because the higher-grade patients have special privileges and exemptions in many matters which may materially affect the rights and the freedom and comfort of the patient. On the other hand, the municipal hospital recognises no obligation to accept paying patients of the higher classes, and will only accept them when there is room.

### Legal Obligations Between Hospital and Patient.

The municipality disclaims all liability for any damage caused to a patient through negligence; and, by treating the relations between the hospital and the patient as a private agreement, contracts itself outside the ordinary law which enacts the exact contrary. Authority is taken to expel patients for gross offences and to prevent the admission of an expelled patient to other municipal hospitals except in special cases. The previous consent of a legal representative or other relative is necessary before an operation on a patient, but this consent is assumed unless objection be raised to the treatment within two days from the date of the notice of such operation being issued. In urgent cases a shorter delay is permitted. In the case of death the friends have to remove the body within twenty-four hours, or it may be delivered to the mortuary. Dissection is allowed where a question of scientific interest or confirmation of the diagnosis is entailed, unless within twenty-four hours of the notice of death the friends lodge objection. In exceptional cases a post-mortem may be made in less than twenty-four hours after death. In some of the most recently erected hospitals the pathological building contains accommodation for the treatment of bodies where the friends refuse leave for a post-mortem. Should the friends not put in an appearance within three days of the death the hospital may arrange with an undertaker and pay for the cost out of insurance money or other resources of the patient in the hands of the hospital authorities. Otherwise the patient is buried as a pauper by the poor-law authorities.

(*To be continued.*)

